# Executive functions, mental health, and quality of life in healthy older adults

**DOI:** 10.1590/1980-5764-DN-2024-0156

**Published:** 2024-11-11

**Authors:** Cássia Elisa Rossetto Verga, Gabriela dos Santos, Tiago Nascimento Ordonez, Ana Paula Bagli Moreira, Laydiane Alves Costa, Luiz Carlos de Moraes, Patrícia Lessa, Neide Pereira Cardoso, Gustavo Domingos França, Ambrósio Ferri, Beatriz Aparecida Ozello Gutierrez, Henrique Salmazo da Silva, Sonia Maria Dozzi Brucki, Thais Bento Lima da Silva

**Affiliations:** 1 Universidade de São Paulo, Escola de Artes, Ciências e Humanidades, Graduação e Pós-Graduação em Gerontologia, São Paulo SP, Brazil.; 2 Universidade de São Paulo, Faculdade de Medicina, Hospital das Clínicas, Grupo de Neurologia Cognitiva e do Comportamento, São Paulo SP, Brazil.; 3 Instituto Supera de Educação, São José dos Campos SP, Brazil.; 4 Universidade Católica de Brasília, Programa de Pós-Graduação, Gerontologia, Brasília DF, Brazil.

**Keywords:** Frail Elderly, Mental Health, Quality of Life, Executive Function, Cognitive Aging, Idoso Fragilizado, Saúde Mental, Qualidade de Vida, Função Executiva, Envelhecimento Cognitivo

## Abstract

Population aging brings about numerous challenges, particularly concerning cognitive health. In this context, socioeconomic factors such as education have received special attention due to their role in brain health.

**Objective:**

The aim of this study was to describe the performance of executive functions, mental health variables, and quality of life among healthy older adults in relation to their level of education.

**Methods:**

A descriptive, cross-sectional study was conducted with participants aged 60 or older. The assessment protocol included Addenbrooke’s cognitive examination and FAS, trail-making tests A and B, Control, Autonomy, Self-Realization, and Pleasure Scale (CASP-19), as well as the depression, anxiety, and stress scale.

**Results:**

Significant differences were found in the performance of executive functions among older adults with higher levels of education. However, mental health and quality of life variables were only related to participants’ age.

**Conclusion:**

The study showed that mental health and quality of life are not influenced by participants’ level of education but are instead strongly correlated with age.

## RESUMO

O envelhecimento populacional traz consigo inúmeros desafios, especialmente no que diz respeito à saúde cognitiva. Nesse contexto, fatores socioeconômicos como a educação têm recebido atenção especial em razão do seu papel na saúde cerebral.

**Objetivo:**

Este estudo teve como objetivo descrever o desempenho das funções executivas, variáveis de saúde mental e qualidade de vida entre idosos saudáveis em relação ao seu nível de educação.

**Métodos:**

Foi realizado um estudo descritivo e transversal com participantes de 60 anos ou mais. O protocolo de avaliação incluiu o Exame Cognitivo de Addenbrooke e Teste de Fluência Verbal (FAS), Testes de Trilhas A e B, Escala de Controle, Autonomia, Autorrealização e Prazer (CASP-19), bem como a Escala de Depressão, Ansiedade e Estresse.

**Resultados:**

Foram encontradas diferenças significativas no desempenho das funções executivas entre idosos com níveis mais elevados de educação. No entanto, variáveis de saúde mental e qualidade de vida estavam relacionadas apenas à idade dos participantes.

**Conclusão:**

O estudo mostrou que a saúde mental e a qualidade de vida não são influenciadas pelo nível de educação dos participantes, mas sim fortemente correlacionadas com a idade.

## INTRODUCTION

The aging of the Brazilian population, which began in the 1950s, can be regarded as an effect of demographic change, although this has not occurred uniformly across the country. This process is a key issue for public health in Brazil, given the specific profile of diseases, disabilities, and consequences inherent to this age group. Thus, methods and approaches to preserve and improve health that cater to the needs of older people are needed^1^.

However, with growing life expectancy, population aging has been accompanied by numerous discoveries and challenges in a variety of areas, including cognitive health. At present, roughly 50 million people are living with dementia worldwide, a figure predicted to triple by 2050. More recently, socioeconomic factors such as education have attracted research attention in this process due to their role in brain health^2^.

Education was identified as an important protective factor against the risk of developing dementia. According to the study by Thow et al.^3^, education in early childhood is associated with a lower risk of dementia in adulthood, suggesting that education represents a neuroprotective factor for cognitive domains, such as executive functions (EFs). EFs are defined as a set of skills to establish goals and plan and execute plans effectively. This cognitive domain is essential for an independent and socially active life and has been associated with quality of life (QoL) and mental health in aging. In this sense, the question to be investigated in this study is whether EFs vary at different levels of education? Or would they be associated with age and educational level?

As described in the literature, individuals who have a higher level of education tend to present less cognitive impairment in the aging process. This is due to the accumulation of cognitive reserve, acquired through different forms of learning throughout life. In this sense, it is expected that better performance in EFs is associated with a greater number of years of schooling^4^.

Still, age represents a risk factor for more pronounced impairments in relation to certain cognitive abilities, such as EF. Despite this, through cognitive training, older people can present good cognitive performance. It is also noted that cognitive interventions can benefit mood, increase socialization, and help with better performance in everyday tasks, which reduces the presence of depressive symptoms^5^.

Brazil is set to experience a rise in the prevalence of psychosomatic and degenerative diseases, such as dementia and depression, as a result of the global trend in human aging. These diseases are triggered by a multifactorial process, which reduces the QoL and functional capacity of older people. According to Pereira et al.^6^, these diseases are more prevalent among older adults, and, by 2030 and 2050, an estimated 66 million and 131 million people, respectively, will suffer from these psychiatric disorders^6^.

Cognition allows knowledge to be acquired and provides the ability to understand information, memorize, and perform activities^7^. EFs, responsible for planning and executing tasks, are one of the main domains affected by cognitive aging. Although EFs decline naturally, this may occur faster than expected, depending on the intrinsic (physical and mental) and extrinsic (environmental) characteristics of the individual. Physical and mental abilities, i.e., intrinsic characteristics, are related to functional capacity, which enables autonomy and independent living to be maintained. This capacity strongly impacts the QoL and well-being of older individuals^7,8^.

In this way, recognizing the positive and negative factors associated with cognitive performance, in this case, specifically EFs, can help in making strategic decisions in relation to the aging of the population, for example, by strengthening teaching-learning actions throughout life and implementing policies to encourage the maintenance of educational activities in adulthood and old age.

In the study by Lopes et al.^9^, the authors carried out a systematic review of the literature on training EFs in older adults. The study underlined the importance of EFs in the cognitive processes that manage people’s lives and contribute to healthy aging. The review involved a selection of studies of older adults aged 60–85 years, where the average number of training sessions was 12, most administered on a weekly basis. The results showed that, after the cognitive training intervention, the older participants in the training group showed improved cognitive performance, higher rates of psychological well-being, and better perceived QoL^7^.

In another innovative study, Rodrigues et al.^10^ investigated the effects of age and education, as well as their interactions, on the performance of adults on the Brief Neuropsychological Assessment Battery (NEUPSILIN), evaluating place–time orientation, attention, perception, memory, language, and EFs. The sample comprised 672 healthy adults (age 19–90 years), stratified into three groups according to education (1–4, 5–8, and ≥9 years of formal education) and into four groups according to age (19–39, 40–59, 60–75, and 76–90 years).

Significant results and greater performance in the assessed tasks were found for the schooling variable, but not for age. The authors noted that stimuli in the academic setting likely promoted the development of metacognitive skills, such as learning arithmetic and language skills, which helped participants perform better on neuropsychological tasks. However, they observed that during the aging process, particularly in old age, education impacts EF heterogeneously^10^.

In this respect, individuals with unfavorable socioeconomic factors, such as education, sex, and income, can be negatively impacted in terms of cognitive development during childhood and adolescence, influencing cognitive performance in adulthood and later life. This influence on cognitive development, in turn, can negatively impact cognitive health, affecting socioeconomic status over the lifespan^2^.

Although EFs are essential for the development and preservation of an autonomous and independent life and there are changes in their functioning throughout the life cycle, few studies seek to understand the specific associations of this cognitive function with other variables. Many investigations are related to mnemonic strategies^11^.

Therefore, the objective of the present study was to describe the performance in EF, mental health variables, and QoL of healthy community-dwelling older adults in relation to the level of education of the participants.

## METHODS

A descriptive cross-sectional study of older adults aged ≥60 years was conducted. Participants were recruited from community centers for older people and from retiree associations in São Paulo city.

As for the inclusion criteria, 207 older adults aged 60 years or older, of both sexes, residing in the city of São Paulo and linked to Centers for Living Together for the Older or Associations of Retirees and Pensions were recruited.

The following exclusion criteria were applied: older individuals aged <60 or >90 years presenting visual, auditory, or motor deficits precluding comprehension of instructions and performing of cognitive tasks; other uncontrolled clinical diseases such as systemic arterial hypertension (SAH) and diabetes mellitus (DM), psychiatric disorders, such as severe depression, schizophrenia, and bipolar disorder, among others; clinical evidence or previous neuroimaging exams disclosing signs of vascular disease; and individuals diagnosed with dementia. This information was obtained through self-reporting by interviewees.

In addition, participant recruitment and evaluation took place between February and March 2022. The present study is part of a larger research project with a database holding baseline data on neuropsychological assessments, namely: *A eficácia de um programa de estimulação cognitiva com componentes multifatoriais na cognição e em variáveis psicossociais de idosos sem demência e sem depressão: um ensaio clínico randomizado e controlado* (*Effectiveness of a cognitive stimulation program with multifactorial components on cognition and on psychosocial variables of older adults with no dementia or depression: a randomized clinically-controlled trial*).

A 90-min assessment with data collection on neuropsychological variables was performed for the present study. All participants signed the Free and Informed Consent Term guaranteeing the anonymity and confidentiality of the data and declaring the right to withdraw from the study at any time. The procedures used to assess study participants included the following neuropsychological assessment protocols:

- Addenbrooke’s Cognitive Examination-Revised (ACE-R): A brief battery measuring six cognitive domains (orientation, attention, memory, verbal fluency, language, and visuospatial ability). The tool is useful for detecting dementia and mild cognitive impairment^12,13^.
- Verbal Fluency Test (FAS category): This test measures the ability to name as many words as possible, beginning with the letters “F,” “A,” and “S” in 1 min for each letter. This test evaluates semantic memory, EFs, and language^14^.
- The Trail-Making test consisted of two parts (Trail-Making A and B): The task consisted of joining numbers and letters alternately (1-A, 2-B, 3-C, etc.). The test measures attention, sequencing, mental flexibility, visual searching, and motor function^15^.
- The Control, Autonomy, Self-Realization, and Pleasure (CASP-19) scale: A 19-item scale measuring perceived QoL in subjects aged ≥55 years^16^.
- The CASP-19 is based on four psychological needs defined by Maslow to assess QoL, namely, control, autonomy, self-realization, and pleasure, which capture the active and reflective processes of being human and freely participating in social life^17^.
- Depression, Anxiety, and Stress Scale (Short Form DASS21): This scale was developed to measure and differentiate symptoms of anxiety and depression. The scale is based on three models that group symptoms of anxiety and depression. The original scale contained 42 items, but the short version of 21 items with questions about depression, stress, and anxiety was used in the present study^18^. The sample profile is summarized in tables containing frequency and descriptive statistics with location and dispersion measures, together with absolute and relative frequencies.

The Kolmogorov-Smirnov test showed a non-normal distribution, and, therefore, non-parametric tests were applied. Thus, the chi-square test was used to compare categorical variables for the diagnostic groups, the Mann-Whitney U-test was applied to compare continuous and ordinal data, the relationship between continuous and ordinal data was analyzed using the Spearman correlation test, while logistic regression was employed for the analysis of multivariate association.

Data were keyed into Google Forms and stored on Google Sheets. All statistical analyses were carried out using the JASP computer software program^19^. The level of significance (null hypothesis rejection) adopted for statistical tests was 5%, i.e., a p-value <0.05.

The present study was approved by the Ethics Committee for Research involving Humans of the Hospital das Clínicas da Faculdade de Medicina da Universidade São Paulo (HC–FMUSP), under approval no. 4.357.429.

## RESULTS

Table 1 presents the sociodemographic characteristics of the participants, both overall and stratified by educational level. In the overall sample of 207 participants, 73.43% were female and 26.57% were male. The mean age of the participants was 67.55 years (SD=5.29), with a median age of 67.00 years and a range from 60.00 to 89.00 years. Regarding education, the mean number of years of education was 17.03 (SD=4.64), with a median of 16.00 years and a range from 8.00 to 45.00 years.

**Table 1:** Sociodemographic variables of participants in general and stratified.

Variable		Overall		Group I		Group II		p-value
		n=207	%	n=97	%	n=110	%	
Sex	Female	152	73.43	64	65.98	88	80.00	0.023*
	Male	55	26.57	33	34.02	22	20.00	
Age	Mean (SD)	67.55 (5.29)		66.93 (5.30)		68.09 (5.24)		0.065^†^
	Median	67.00		66.00		67.50		
	Min–Max	60.00–89.00		60.00-81.00		60.00–89.00		
Education	Mean (SD)	17.03 (4.64)		20.64 (4.04)		13.85 (2.12)		<0.001^†^
	Median	16.00		20.00		15.00		
	Min–Max	8.00–45.00		17.00–45.00		8.00–16.00		
Marital status	Married	101	48.79	50	51.55	51	46.36	0.494*
	Divorced	32	15.46	17	17.53	15	13.64	
	Single	46	22.22	20	20.62	26	23.64	
	Widowed	28	13.53	10	10.31	18	16.36	
Are you retired or a pensioner?	Yes	176	85.02	81	83.51	95	86.36	0.553*
	No	31	14.98	16	16.49	15	13.64	

Abbreviations: SD, standard deviation.

Note: *χ^2^ test;

^†^Mann-Whitney U-test for independent samples.

When stratified by educational level, Group I (highly educated) consisted of 97 participants, while Group II (less educated) consisted of 110 participants. The proportion of females was slightly higher in Group II (80.00%) compared to Group I (65.98%). Group I had a slightly lower mean age (66.93 years, SD=5.30) compared to Group II (68.09 years, SD=5.24). Additionally, Group I had a higher mean level of education (20.64 years, SD=4.04) compared to Group II (13.85 years, SD=2.12).

Descriptive statistics with measures of central tendency and dispersion (mean, standard deviation, minimum, median, and maximum) for the variables analyzed are presented in Table 2.

**Table 2:** Participants’ general performance in verbal fluency, attention, and quality of life tests.

Variables	n	Mean	SD	Minimum	Median	Maximum
Addenbrooke’s fluency	207	10.86	1.84	5.00	11.00	14.00
Verbal Fluency Test category (FAS)						
F (Total)	207	14.46	3.84	4.00	14.00	26.00
A (Total)	207	13.30	3.88	4.00	13.00	24.00
S (Total)	207	13.43	3.74	4.00	14.00	26.00
Time trail-making tests A	207	47.42	16.47	21.00	44.00	118.00
Trail-making tests A errors	207	0.27	0.68	0.00	0.00	5.00
Time trail-making tests B	207	107.43	61.31	47.00	93.00	595.00
Trail-making tests B errors	207	1.05	1.59	0.00	0.00	10.00
CASP19 control	207	9.79	1.53	5.00	10.00	12.00
CASP19 autonomy	207	11.13	2.14	5.00	11.00	15.00
CASP19 pleasure	207	12.45	2.30	5.00	13.00	15.00
CASP19 self-realization	207	10.04	2.90	3.00	10.00	15.00
CASP19 total	207	43.41	7.21	23.00	43.00	57.00
DASS21 depression	207	3.03	4.26	0.00	2.00	26.00
DASS21 anxiety	207	3.35	4.10	0.00	2.00	26.00
DASS21 stress	207	9.82	6.34	0.00	10.00	34.00
DASS21 total	207	16.20	11.39	0.00	14.00	62.00

Abbreviations: SD, standard deviation; ACE-R, Addenbrooke’s Cognitive Examination; Verbal Fluency Test category (FAS); Trail: Making Test, Trail Test-Trails A and Trails B; CASP19, Control, Autonomy, Self-realization, and Pleasure Scale; DASS21, Depression, Anxiety, and Stress Scale. 83 107 111 115 143 147 151 155 51 55 59 63 47 39 35 31 27.

Results of Mann-Whitney U-tests, used to compare scores on verbal fluency, Trail-Making, CASP19, and DAS21 tests between Groups I and II, are given in Table 3. In the present study, the hypothesis was tested that Group I participants would have superior performance for outcomes measured by the ACE-R, Verbal Fluency FAS category, Trail-Making Tests A and B, CASP19, and DASS21 tests. However, results revealed only differences in the Verbal Fluency category for letter F (p=0.010) and in the Trail-Making B test for time (p=0.030), with better performance observed in Group I.

**Table 3:** Participants’ performance in verbal fluency, attention, and quality of life tests, stratified by education.

Variables	Group	n	Mean	SD	Minimum	Median	Maximum	p-value
Addenbrooke’s fluency	Grupo I	97	11.12	1.77	6.00	11.00	14.00	0.072
	Grupo II	110	10.63	1.89	5.00	11.00	14.00	
F (Total)	Grupo I	97	15.20	3.62	8.00	15.00	24.00	0.010
	Grupo II	110	13.81	3.94	4.00	14.00	26.00	
A (Total)	Grupo I	97	13.61	3.95	4.00	14.00	24.00	0.278
	Grupo II	110	13.04	3.81	4.00	13.00	22.00	
S (Total)	Grupo I	97	13.47	4.00	4.00	14.00	22.00	0.507
	Grupo II	110	13.39	3.51	6.00	13.00	26.00	
Time trail-making tests A	Grupo I	97	45.40	14.61	25.00	42.00	104.00	0.112
	Grupo II	110	49.20	17.83	21.00	46.00	118.00	
Trail-making tests A errors	Grupo I	97	0.29	0.76	0.00	0.00	5.00	0.944
	Grupo II	110	0.25	0.60	0.00	0.00	3.00	
Time trail-making tests B	Grupo I	97	98.90	52.01	47.00	84.00	471.00	0.030
	Grupo II	110	114.96	67.82	52.62	103.50	595.00	
Trail-making tests B errors	Grupo I	97	1.02	1.73	0.00	0.00	10.00	0.320
	Grupo II	110	1.08	1.46	0.00	1.00	7.00	
CASP19 control	Grupo I	97	9.87	1.49	5.00	10.00	12.00	0.460
	Grupo II	110	9.73	1.57	5.00	10.00	12.00	
CASP19 autonomy	Grupo I	97	11.28	2.11	5.00	11.00	15.00	0.339
	Grupo II	110	10.99	2.17	6.00	11.00	15.00	
CASP19 pleasure	Grupo I	97	12.58	2.19	5.00	13.00	15.00	0.578
	Grupo II	110	12.35	2.39	5.00	12.50	15.00	
CASP19 self-realization	Grupo I	97	10.01	2.88	3.00	10.00	15.00	0.832
	Grupo II	110	10.06	2.93	3.00	10.00	15.00	
CASP19 total	Grupo I	97	43.73	6.99	23.00	44.00	57.00	0.495
	Grupo II	110	43.13	7.41	27.00	43.00	57.00	
DASS21 depression	Grupo I	97	2.64	4.08	0.00	2.00	26.00	0.117
	Grupo II	110	3.38	4.40	0.00	2.00	26.00	
DASS21 anxiety	Grupo I	97	3.09	3.70	0.00	2.00	20.00	0.441
	Grupo II	110	3.58	4.43	0.00	2.00	26.00	
DASS21 stress	Grupo I	97	9.94	6.21	0.00	10.00	26.00	0.725
	Grupo II	110	9.71	6.47	0.00	10.00	34.00	
DASS21 total	Grupo I	97	15.67	10.81	0.00	14.00	52.00	0.540
	Grupo II	110	16.67	11.92	0.00	14.00	62.00	

Abbreviations: SD, standars deviation; CASP-19, control, autonomy, self-realization and pleasure; DASS21, depression, anxiety and stress scale.

The speed of completion of Trail-Making test B was faster in Group I, with a mean of 98.90 s, compared with Group II with 114.96 s, whose participants took longer to execute and resolve the same task (Table 3). Comparisons of scores for the variables ACE-R, verbal fluency categories A and S, Trail-Making A for time and error, and Trail-Making test B for errors, CASP19, and DASS21 tests revealed no group differences on the Mann–Whitney U-test.

Table 4 illustrates the Spearman correlation between age and education, as well as other study variables. The table displays the correlation coefficient (rho) and the corresponding p-values for each pair of variables. For age and education, there is a significant inverse correlation (rho=-0.21, p=0.002), indicating that as age increases, education tends to decrease or the opposite.

**Table 4:** Spearman correlation between age and education and other study variables.

Variable	Age		Education	
	rho	p-value	rho	p-value
Age	–	–	-0.21	0.002
Education	-0.21	0.002	–	–
Verbal fluency (Addenbrooke’s)	-0.11	0.100	0.15	0.030
Verbal fluency F (Total)	-0.05	0.492	0.18	0.008
Verbal fluency A (Total)	-0.04	0.566	0.13	0.056
Verbal fluency S (Total)	-0.03	0.714	0.12	0.093
Time trail-making tests A	0.32	<0.001	-0.18	0.008
Trail-making tests A errors	0.14	0.038	0.01	0.897
Time trail-making tests B	0.30	<0.001	-0.20	0.004
Trail-making tests B errors	0.06	0.384	-0.14	0.044
CASP19 control	-0.21	0.003	0.08	0.227
CASP19 autonomy	-0.10	0.147	0.11	0.106
CASP19 pleasure	-0.26	<0.001	0.06	0.400
CASP19 self-realization	-0.24	<0.001	0.02	0.746
Casp19 total	-0.26	<0.001	0.08	0.230
DASS21 depression	0.18	0.011	-0.09	0.206
DASS21 anxiety	0.10	0.150	-0.05	0.472
DASS21 stress	-0.01	0.928	0.01	0.886
DASS21 total	0.09	0.213	-0.04	0.527

Abbreviations: CASP-19, control, autonomy, self-realization and pleasure; DASS21, depression, anxiety and stress scale.

Note: p-value: Spearman rank order correlations.

Regarding verbal fluency measures, there are varied correlations with age. Verbal Fluency F (Total) shows a moderate positive correlation with age (rho=0.18, p=0.008), while Verbal Fluency (Addenbrooke’s) exhibits a weak negative correlation (rho=-0.11, p=0.100). The other verbal fluency measures (Verbal Fluency A, Verbal Fluency S) show similar weak negative correlations with age. In terms of the Trail-Making Tests, both Trail-Making test A time and Trail-Making test B time show strong positive correlations with age (rho=0.32, p<0.001 and rho=0.30, p<0.001, respectively). This suggests that as age increases, the time taken to complete these tests also increases. Additionally, Trail-Making test A errors show a weak positive correlation with age (rho=0.14, p=0.038).

Among the CASP19 domains, there is a significant inverse correlation between age and CASP19 Control (rho=–0.21, p=0.003), CASP19 Pleasure (rho=–0.26, p<0.001), and CASP19 Self-Realization (rho=–0.24, p<0.001). This indicates that as age increases, perceptions of control, pleasure, and self-realization tend to decrease.

Finally, for the DASS21 questionnaire, there is a weak positive correlation between age and DASS21 Depression (rho=0.18, p=0.011), indicating that higher age is associated with greater severity of depressive symptoms. Other domains of DASS21 (Anxiety, Stress, and Total) show weak or no significant correlations with age.

The final analysis included logistic regression to determine the effects of EFs, QoL, and sociodemographic variables (sex, age, marital status, and retirement) on the probability of participants having a high level of education (i.e., belonging to Group I). The logistic regression model was statistically significant. Thus, it was found that a high level of education (>16 years of formal education) was associated with a greater probability of achieving higher performance on the verbal fluency test (category F) and of being male (Table 5).

**Table 5:** Logistic regression to predict what is associated with high education in the studied sample.

Model	Parameter	Estimates	Standard error	z	Wald Test	df	p-value
					Wald Statistics		
1	(Intercept)	0.126	0.139	0.903	0.815	1	0.367
2	(Intercept)	1.536	0.571	2.689	7.231	1	0.007
	F	-0.097	0.038	-2.554	6.524	1	0.011
3	(Intercept)	1.771	0.591	2.997	8.979	1	0.003
	F	-0.100	0.039	-2.580	6.656	1	0.010
	Sex (Male)	-0.747	0.326	-2.290	5.243	1	0.022
4	(Intercept)	-1.750	1.996	-0.877	0.769	1	0.380
	F	-0.098	0.039	-2.523	6.363	1	0.012
	Sex (Male)	-0.845	0.335	-2.518	6.340	1	0.012
	Age	0.052	0.029	1.830	3.348	1	0.067

Abbreviation: df, degree of freedom.

## DISCUSSION

First, it is important to note that education is one of the main protective factors against the risk of developing dementia conditions. According to the literature, education should be considered a key element where, as cited by Thow et al.^3^, early life education is associated with a reduced risk of dementia in later life, suggesting that education may act as a neuroprotective factor of cognitive function.

Therefore, the primary objective of the present study was to elucidate EF performance, mental health variables, and QoL in healthy community-dwelling older adults and determine the potential association of these variables with educational level.

Based on this line of reasoning, according to the study findings, it can be argued that physical and mental abilities, i.e., intrinsic characteristics, demonstrate a significant association with functional capacity. This capacity supports autonomy and independence and, thus, significantly impacts QoL and well-being of older individuals^7,8^.

The hypothesis tested in the present study holds that performance on the tests would be superior among participants with higher levels of education, a predictor for preserved cognition, enhanced QoL, and a factor protecting against the development of dementia. The study results revealed that the higher the educational level of participants, the better their performance on verbal fluency tests (ACE-R, F, and A) and Trail-Making Tests A and B, supporting the initial hypothesis and corroborating the results of previous studies by Oliveira et al.^7^ and by Karsch^8^.

Individuals who have more years of education tend to perform better on cognitive tests, and this disparity can be explained by a combination of biological, psychological, and social factors. Cognitive reserve, for example, is a key concept in this context. Individuals with greater education generally have access to a richer educational environment, which contributes to the development of more complex and resilient neural networks. This cognitive reserve can help compensate for the effects of aging or possible brain damage caused by pathologies such as dementia, allowing these people to maintain superior cognitive performance even in the face of challenges^20^.

Psychological well-being, in turn, also plays an important role in the cognitive performance of elderly people and is associated with education. People with more years of education tend to have greater self-esteem, a sense of control, and more effective coping skills, which can contribute to better cognitive performance. Education can also provide people with cognitive and emotional tools to deal with stress and adversity, thereby reducing negative impacts on cognitive functioning^21^.

Social relationships contribute significantly to both cognitive performance and QoL. Social support from these networks can promote emotional and psychological well-being, reducing stress and anxiety, factors that can harm the performance of cognitive skills^22^.

The study by Costa et al.^23^ investigated a heterogeneous group of older adults, analyzing verbal fluency performance in physically active older individuals with low education (1–4 years of formal education). As a measure of verbal performance, researchers applied the verbal fluency task to the animal category. Results revealed a good level of performance by participants, despite their low level of education. In spite of this significant finding, the authors suggested similar studies controlling for education be conducted, as carried out in the present study.

This lack of control over educational level was evident in the study by Lima-Silva^24^, in which the authors stratified the older participants into physically active and nonactive groups. The study showed that the physically active group had better performance on verbal fluency tests than the non-physically active group. In the cited study, the verbal fluency test — animals category — was applied as per Costa et al.^23^, but there was no restriction on the educational level of participants.

In the study by Silva et al.^25^, participants were grouped according to educational level, and sociogeographic aspects were also analyzed. The results showed higher scores for verbal fluency among highly educated individuals, irrespective of geographic location.

The observations of the authors have a bearing on the approach adopted in the present study. However, as suggested by Costa et al.^23^, the present study used education as a control factor, but the physical activity variable (active or nonactive) was not evaluated.

Nevertheless, the present study yielded theoretical evidence that can inform future studies exploring this research problem. In the present study sample, no associations or correlations were found between education and mental health variables (depression, anxiety, and stress) or with QoL (CASP19).

These results suggest that mental health and QoL were not influenced by the educational level of participants but were strongly correlated with age, consistent with the findings of Thow et al.^3^. However, lower age was associated with greater perceived QoL, faster completion of the Trail-Making test, fewer depressive symptoms, better performance on the fluency test, and a greater number of years of education.

The literature highlights the determinants responsible for QoL in aging and old age. The perception of a higher QoL is associated with the active participation of elderly people in physical, social, and economic activities, factors that correspond to the concept of active aging^26^. It is therefore worth questioning whether older elderly people experience less active aging compared to younger elderly people.

In this follow-up, education had no direct influence on the constructs of QoL or mental health but exerted an indirect effect when associated with age. Considering this scenario, recent data from the sample of the Estudo Longitudinal da Saúde do Adulto — Longitudinal Study on Health in Adults (ELSA-Brasil) showed that socioeconomic status in early life continues to impact cognitive performance later in adulthood, irrespective of educational level or socioeconomic status in older age.

The study by Socoloski et al.^1^ identified barriers to engaging in physical activity among Brazilian older adults, as a potential factor for developing chronic diseases. Of the factors identified, individuals aged >60 years reported that fear of injury or falls when performing physical activity was a barrier to engaging in these activities. Based on these findings, socioeconomic, demographic, and educational aspects are also noted, factors that are statistically low.

Drawing on these assumptions, Pereira et al.^6^ corroborated the findings of the above-cited author, confirming an association of diagnostic factors with the incidence of neurodegenerative diseases in aging individuals. The authors underscored the importance of education for health and the adoption of a physically active lifestyle to promote healthy aging with enhanced social QoL, particularly physical and mental health.

However, lower age was associated with greater perceived QoL, faster time on the Trail-Making test, fewer depressive symptoms, better performance on the fluency test, and a greater number of years of education. Hence, education exhibited no direct influence on the constructs of QoL or mental health but exerted an indirect effect when associated with age.

In addition to age and other determinants of active aging, psychological aspects influence the QoL considerably. Periods of stress, insecurity, and emotional overload, as experienced during the COVID-19 pandemic, are examples of markers of emotional suffering and consequently worse quality of life^27-29^.

In this context, Marzo et al.^28^ investigated the predictors of physical and mental QoL in elderly residents of six Asian countries. Analysis of data from the 1644 interviewees demonstrated that factors such as age, marital status, income, and social support influenced self-perceptions of QoL.

One of the most important factors for maintaining a good QoL refers to the social relationships established and maintained in old age. In recent years, social networks have been used as a channel for both creating and maintaining emotional relationships. As described by Chen et al., actions to encourage the use and mastery of social networks by the elderly must be encouraged so that these tools enhance the QoL, especially in the affective aspects^30^.

Contributions of the study include the fact that the lack of previous research on the topic of EF performance, mental health, and QoL in healthy older people exploring potential correlations of these variables with education, makes this investigation especially relevant given the importance of health promotion strategies in aging that help maintain autonomy, independence, and QoL in later life.

Professional gerontologists can thus help promote public care policies aimed at maintaining brain health among healthy older individuals, in line with the WHO Global Action Plan on Dementia^31^, which includes seven goals, one of which is to reduce the risk of developing dementia in the years ahead.

In terms of public policies, it is important to promote investments in research and intervention programs aimed at promoting cognitive health in the elderly. This may include initiatives that aim to increase access to lifelong educational and cognitively stimulating activities, such as continuing education courses and cognitive training programs. Policies to encourage healthy lifestyles, such as a balanced diet, regular physical exercise, and control of cardiovascular risk factors, are also essential.

Specific interventions may include cognitive training programs tailored to the needs and interests of older adults, with a focus on maintaining and improving cognitive performance over time. This may involve the use of digital technologies, educational games, and social activities.

In line with the WHO Global Action Plan on Dementia, it is important to include specific targets related to promoting brain health and preventing cognitive decline in healthy older people. This may involve implementing policies and programs that aim to reduce known risk factors for dementia, such as hypertension, diabetes, smoking, and physical inactivity, as well as promoting environments conducive to healthy aging in communities and healthcare institutions.

The study in question has limitations that may affect the interpretation of the results and the generalization of the findings. One of the limitations highlighted is the sample size, which is relatively small. This may compromise the representativeness of the results, making them less generalizable. Furthermore, the presence of heterogeneous groups in terms of sociodemographic factors, such as gender, income, and education, can introduce biases and make it difficult to analyze specific associations between variables.

The heterogeneity of sociodemographic groups can obscure the true effects of the variables under study, since different groups may have different characteristics and social contexts that influence the results. For example, the association between education and cognitive performance may be confounded by the influence of other factors, such as income or access to health services.

Another important limitation is the cross-sectional design of the study, in which exposure and outcome are measured simultaneously. This prevents the inference of causality and limits the ability to establish cause-and-effect relationships between variables. Furthermore, the cross-sectional design may not capture changes over time and underestimate the complexity of the relationships between the variables studied.

Regarding possible biases and measurement errors, it is important to consider that cognitive assessment can be influenced by a variety of aspects, such as emotional state, level of motivation, and familiarity with the test. The lack of standardization in the application of cognitive tests can also lead to inconsistent results and make comparisons between studies difficult.

Given these limitations, it is essential to interpret the study results with caution and recognize their possible restrictions. The generalization of findings to other populations or contexts must be done with caution, taking into account the specific characteristics of the study, such as sample size, group composition, and methodological design. For future research, it is recommended to carry out longitudinal studies with more representative samples and homogeneous groups in relation to the variable of interest, in addition to the use of more robust analysis methods to control potential confounding factors and bias.
